# New System for Digital to Analog Transformation and Reconstruction of 12-Lead ECGs

**DOI:** 10.1371/journal.pone.0061076

**Published:** 2013-04-11

**Authors:** Roshni Kothadia, Walter B. Kulecz, Igor S. Kofman, Adam J. Black, James W. Grier, Todd T. Schlegel

**Affiliations:** 1 National Space Biomedical Research Institute, Houston, Texas, United States of America; 2 Wyle Science, Technology & Engineering Group, Houston, Texas, United States of America; 3 University of Minnesota, Minneapolis, Minnesota, United States of America; 4 North Dakota State University, Fargo, North Dakota, United States of America; 5 NASA Johnson Space Center, Houston, Texas, United States of America; University of Adelaide, Australia

## Abstract

**Introduction:**

We describe initial validation of a new system for digital to analog conversion (DAC) and reconstruction of 12-lead ECGs. The system utilizes an open and optimized software format with a commensurately optimized DAC hardware configuration to accurately reproduce, from digital files, the original analog electrocardiographic signals of previously instrumented patients. By doing so, the system also ultimately allows for transmission of data collected on one manufacturer's 12-lead ECG hardware/software into that of any other.

**Materials and Methods:**

To initially validate the system, we compared original and post-DAC re-digitized 12-lead ECG data files (∼5-minutes long) in two types of validation studies in 10 patients. The first type *quantitatively* compared the total waveform voltage differences between the original and re-digitized data while the second type *qualitatively* compared the automated electrocardiographic diagnostic statements generated by the original versus re-digitized data.

**Results:**

The grand-averaged difference in root mean squared voltage between the original and re-digitized data was 20.8 µV per channel when re-digitization involved the same manufacturer's analog to digital converter (ADC) as the original digitization, and 28.4 µV per channel when it involved a different manufacturer's ADC. Automated diagnostic statements generated by the original versus reconstructed data did not differ when using the diagnostic algorithm from the same manufacturer on whose device the original data were collected, and differed only slightly for just 1 of 10 patients when using a third-party diagnostic algorithm throughout.

**Conclusion:**

Original analog 12-lead ECG signals can be reconstructed from digital data files with accuracy sufficient for clinical use. Such reconstructions can readily enable automated second opinions for difficult-to-interpret 12-lead ECGs, either locally or remotely through the use of dedicated or cloud-based servers.

## Introduction

Most modern electrocardiogram (ECG) machines use built-in analog to digital converters (ADCs) to digitize patients' analog cardiac electrical signals for more efficient analysis, display, storage, printing, and sharing of data. While this common and intuitive method has heretofore been sufficient for most clinical uses, it typically “locks in” the practicing clinician to the often opaque and sometimes proprietary digital format(s) of the specific ECG machine(s) being employed. In contradistinction, and particularly for patients with a difficult-to-interpret 12-lead ECGs wherein the automated diagnosis from the “house machine” may be in question (no automated algorithm being error free), many clinicians might welcome the opportunity to readily obtain one or more additional opinions from other manufacturers' automated interpretive algorithms. Different algorithms for example are sometimes known to have widely varying diagnostic accuracies for common electrocardiographic conditions [1].

If through non-disclosure or other agreements with ECG manufacturers a researcher is given direct digital access into an automated interpretive program for 12-lead ECG that accepts a known digital format, then it is relatively straightforward to convert other known digital formats into that first known digital format to thereby gain access to the interpretive functionality. In principle therefore an “ideal” (albeit still non-universal) means for clinicians to obtain automated second opinions for 12-lead ECGs would involve a fully digital interchange wherein multiple manufacturers would allow for simultaneous digital access to their interpretive functionalities using a common digital format. In practice, however, such interchanges do not exist for the general clinical community, one historical reason being the obstruction created by the manufacturers' naturally competing commercial interests. Thus while the previous starts that have been made toward such interchanges such as those of Bailey et al [2] in the 1970s and of Willems et al [1] in 1990s are of great interest, the fact that such starts have never germinated into a clinically useful, potentially lifesaving tool speaks to the inertia that can be generated when certain commercial forces persist that are not necessarily ideal from a patient-centered medicine standpoint. It was possibly this very non-ideality that in 1984 led Miyahara et al to take a slightly different approach of first collecting digital ECGs and then painstakingly regenerating *analog* signals – one complex at a time by means of magnetic tape and a specially constructed “generator” – that they then re-fed into 10 different interpretive ECG machines available in Japan [3]. However, the methods described by Miyahara et al are today obsolete, were unfortunately very cumbersome, and possibly also involved two serial (and thus clinically redundant) references to Wilson's central terminal (WCT).

Herein we describe a new digital to analog conversion system based on contemporary computer and electrical engineering technology that is readily available. It can reproduce with sufficient precision for clinical use the original analog ECG signals from any 12-lead ECG digital data file or stream of known format, thereby allowing for the complete reconstruction of the original ECG after “re-digitization” within any brand and model of receiving 12-lead ECG machine. It can do this either locally or remotely and without any requirement for manufacturer-adjudicated digital access into the receiving machine, thereby specifically allowing for the transmission of data collected on one manufacturer's ECG machine into that of any other for an automated diagnostic second opinion. Thus the system could be valuable for facilitating – by consensus among algorithms or physician judgment in conjunction with machine interpretation – the ultimately correct interpretation of difficult-to-interpret 12-lead ECGs and rhythms. Moreover because the system also performs its function with full “universality” (something that may never be practically possible for any purely digital interchange), over the short term it's also likely to better foster further improvements to *all* ECG manufacturers' (large and small) automated interpretive programs through provision to those manufacturers of multiple and repeatable input example cases that their diagnostic algorithms currently misdiagnose.

Although the concept of using a digital to analog converter (DAC) to retrieve original analog ECG waveforms is not novel (as described previously [3], plus it has been applied for decades in ECG simulator devices), we are aware of only one commercially available system (LifeSync®, Fort Lauderdale, FL) that presently applies the DAC concept to a patient-care setting. That system, however, utilizes a different type of technology to satisfy a different clinical need – i.e., it is designed to provide hospitalized, ECG-monitored patients with greater freedom of movement and less risk for hospital-acquired infections from otherwise reused and wall-tethered lead wires, certainly laudable goals themselves. Unfortunately the LifeSync DAC does not accept digitized ECG data from any ADC other than LifeSync's own, nor to our knowledge does it transmit digital data to remote locations. Instead, from an electronics standpoint, the LifeSync DAC functions as the “straight pass through” recipient of 9 channels of specially structured digital data (rather than the more reductionist and customary 8 channels) that can only originate from an accompanying LifeSync ADC device. Thus the LifeSync DAC procedure carries with it the absolute requirement not only for the presence of the LifeSync DAC device, but also for the use of the LifeSync ADC and all of its accoutrements during the original data collection.

The DAC system introduced herein is instead designed to begin with digital data, stored or streaming, *collected on any ECG manufacturer's ADC*. It is therefore independent of any particular manufacturer's 12-lead ECG hardware and can thus function in harmony with any 12-lead ECG machine used for data collection. The only requirement is that the digital format utilized by the given data collection machine must be known because that format will typically first undergo a purely digital (software-based) conversion to an optimal, open digital format (provided in Appendix S1) that is specifically designed to optimally reproduce (with the DAC hardware) the original analog ECG signals. Once the original analog signals are reproduced, the system can then move those signals forward into any other manufacturer's ECG machine(s) to be re-digitized (or “reconstructed”) there. Thus automated diagnostic interpretations from multiple manufacturers' ECG machines can be obtained for any ECG data file or stream of known format collected by any other manufacturers' machine(s), either locally or remotely, and with any desired degree of fidelity dependent only on the specifications of the ADCs used for the original and reconstruction data collections.

The prototype system described herein was also specifically designed to expand automated analytical capabilities for 12-lead ECG data collected in certain remote places wherein the mass or volume of the ECG device must be constrained and/or wherein interpretive expertise is limited at the remote location. For example for 12-lead ECG data arriving from space or from remote terrestrial environments such as mobile military units, oil platforms or mountaineering, polar or other expedition areas.

## Methods

### The methodological problem

**Figure 1:**
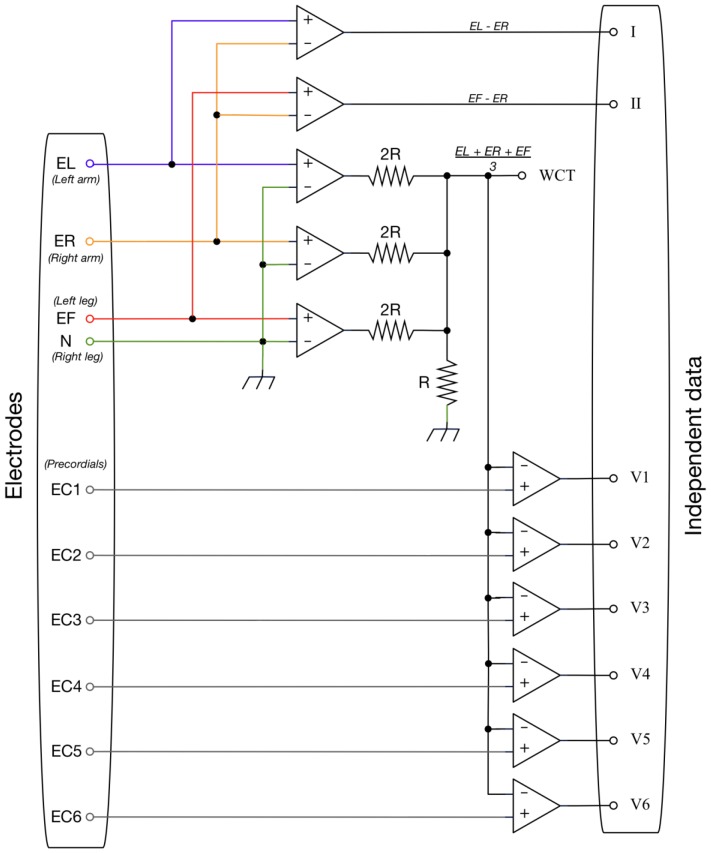
Generalized functional diagram for a typical 12-lead ECG system.

When collecting a standard 12-lead ECG, 10 electrodes placed on the patient are used to obtain (typically) up to 9 different analog voltages. These voltages are then most commonly stored digitally as 8 independent data channels (i.e., typically as channels that are equivalent to leads I and II plus leads V_1_–V_6_ as referenced to WCT [4]). Now if one defines the original 10 electrodes as follows: left arm electrode = EL; left leg electrode = EF; right arm electrode = ER; right leg electrode = N (reference neutral) and chest electrodes = EC_i_ (where i = 1–6), then the 8 independent data channels would most commonly be expressed as:
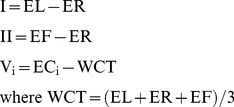
Thus the methodological problem the system must solve is as follows: How can one begin with 8 independent data channels in the original digital data and yet drive at least 9 DAC channels uncoupled from WCT (because the final receiving ECG machine is itself expected do such coupling) to produce these same data channels (leads I, II and V_1_–V_6_) at the receiving ECG machine? In other words how can one do this as if the channels ultimately outputted by the DAC had come from the usual 10 electrodes on a patient, and with the right leg electrode input remaining as DAC common? Figure 1 expresses this problem graphically by showing a generalized functional diagram for a typical 12-lead ECG system, but ignoring (as defined) the non-independent leads III, aVR, aVL and aVF.

### A methodological solution

For any 12-lead ECG system that uses a digital file format wherein the chest electrodes are referenced not to WCT, but instead to the right arm electrode (i.e., to ER, thereby producing CR_i_ instead of V_i_ chest lead data), the following applies:
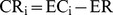
Moreover, if in a DAC that is also associated with (receives digital data from) such a system, a “zero” voltage is imposed upon its right arm electrode input (i.e., ER = 0), then from that DAC: 
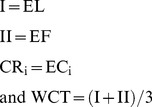
Therefore, if the following conditions are assigned to the DAC, they should ultimately produce, on any ultimately receiving (re-digitizing) 12-lead ECG machine, the desired I, II, and V_1_–V_6_ data signals:
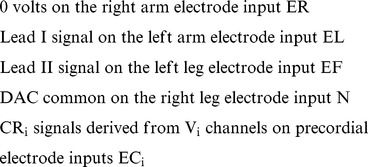



At least two other aspects of the above system are of interest. First, this system type where the chest electrodes are referenced not to WCT, but instead to the right arm electrode, was originally favored not only by Einthoven himself [5], but also by others even after the introduction of WCT [6], [7]. Second, algebraically it is also possible to accomplish the same fundamental end point through a digital format wherein all other electrodes are referenced to the left arm electrode while a zero voltage is simultaneously imposed on the DAC left arm electrode input, or through a digital format wherein all other electrodes are referenced to the left leg electrode while a zero voltage is simultaneously imposed on the DAC left leg electrode input.

### Optimized file format, hardware and software configuration, and data processing procedures

**Figure 2:**
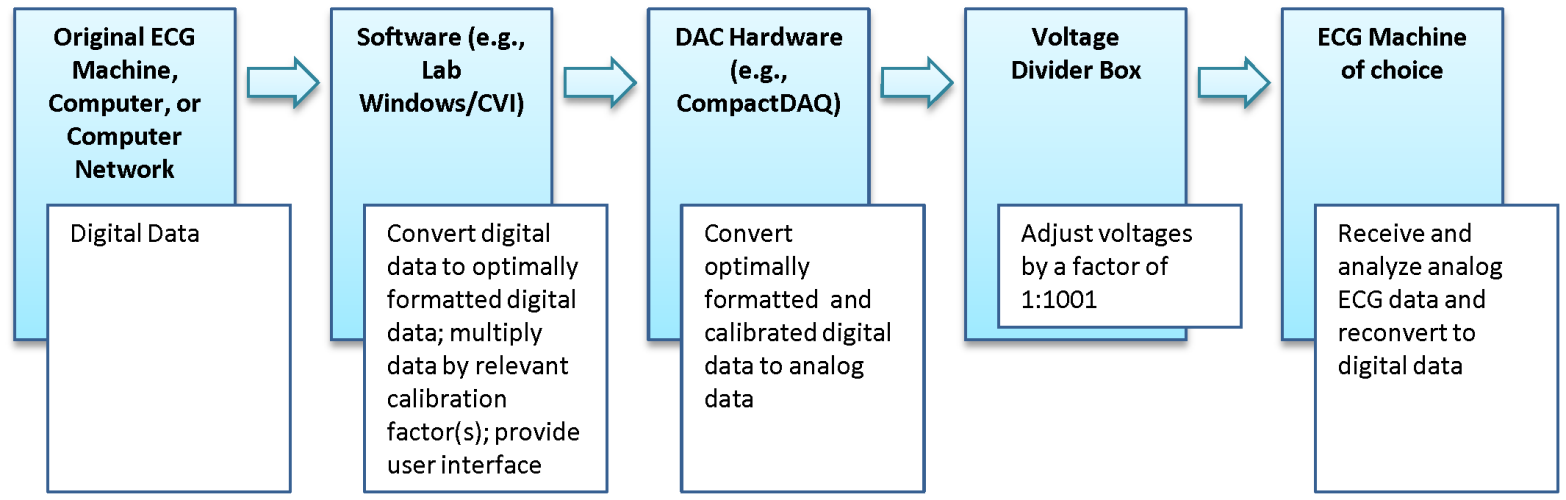
Schematic summarizing all stages of ECG data processing by the system.

The format of any digital data inputted into the preferred (“right arm zeroed”) DAC described above must be compatible with that DAC's specific characteristics. Such an optimized data format, into which all other digital formats must therefore be converted before use with the DAC, is further detailed in Appendix S1. The specific hardware and software configuration of our prototype, including the details of how it currently processes ECG data, is outlined in Appendix S2. Figure 2 also provides a summary overview of all data processing procedures.

### Initial validation studies

For the initial validation studies described herein, we used ten 12-lead ECG data files, each between 5 and 10 minutes in length, collected from five healthy and five diseased patients, respectively, who had given their written informed consent for participation in a larger study previously approved by the Johnson Space Center Institutional Review Board [8]. Their data were originally collected using a high-fidelity 12-lead PC-ECG device (Cardiax, IMED Ltd., Budapest, Hungary). The 12-lead ECGs were clinically normal in each of the five healthy patients chosen at random, whereas in the five diseased patients, the following electrocardiographic conditions were respectively selected (from affected individuals also chosen at random) to include a range of electrocardiographic pathologies: *1*) coronary artery disease without prior myocardial infarction and with normal QRS interval; *2*) coronary artery disease with prior myocardial infarction (i.e., ischemic cardiomyopathy) but with normal QRS interval; *3*) non-ischemic (dilated) cardiomyopathy with normal QRS interval; *4*) left bundle branch block of uncertain etiology; and *5*) right bundle branch block of uncertain etiology.

Two types of validation studies were performed to compare the original digital ECG data to their reconstructed (i.e., after DAC and repeat ADC) counterpart data. The first type *quantitatively* compared the total-waveform voltage differences between the original and reconstructed data while the second type *qualitatively* compared the automated electrocardiographic (i.e., clinical) diagnostic statements generated by the original versus reconstructed data.

### Quantitative validation

A MATLAB-based script was written to superimpose the data in the original and reconstructed files for each subject by using the corresponding R-wave fiducial point locations in the files to align the corresponding waveforms. For this purpose the original R-wave fiducial point locations were obtained directly, within exported files along with the raw data, from the hardware manufacturer's (Cardiax's) commercial software itself. Each test file had 250 to 500 PQRST complexes within a 5 to 10-minute data epoch. For each PQRST complex, a region about the R-wave fiducial point was used to define a window encompassing the PQRST segments with minimal amounts of pre-P and post-T baseline. The data in this window were linearly de-trended and the original versus reconstructed waveforms were overlaid and shifted to minimize the root mean square (RMS) difference. The standard deviation was used as a proxy because detrending alone ensures a near zero mean but not a perfectly zero mean. An average RMS difference estimate across all beats was then calculated for each channel in each patient, as was an overall average RMS difference for all channels combined. This same process was performed twice: once after having used the same model of ECG machine (Cardiax ADC; 1000 samples/s/channel) to collect the reconstructed (re-digitized) data that had also been used to collect the original data; and once after having used a different manufacturer's ECG machine (BT12 ADC, CorScience, Erlangen, Germany; 500 samples/s/channel) to collect the re-digitized data.

### Qualitative (automated clinical diagnostic) validation

A more qualitative (clinical) validation was also performed to further validate system performance. Specifically, the automated diagnostic statements, produced by commercial electrocardiographic software for the data within the first ∼15 seconds in the original files, were compared in each case to the automated diagnostic statements produced for the same data in the post-DAC re-digitized files. Such analyses of potential changes in automated diagnostic statements were in turn performed in three separate ways: *1*) by using the automated diagnostic program native to the Cardiax software program when a Cardiax ECG machine (ADC) had been used to collect both the original and re-digitized data; *2*) by using the well-validated Leuven automated diagnostic algorithm [1] (see Program 16 in reference 1) for both the original data and the re-digitized data when a Cardiax ADC had been used to collect both the original and re-digitized data; and *3*) by again using the Leuven automated diagnostic algorithm for both the original data and for the re-digitized data when a Cardiax ADC had been used to collect the original data but a CorScience BT12 ADC the re-digitized data.

## Results

### Quantitative validation: voltage comparison results

Table 1 shows the estimated RMS difference values for each of the 8 independent ECG channels (PQRST) when the same model of ECG machine (Cardiax ADC) that had been used to collect the original data was also used to collect the re-digitized data. Under these circumstances, the grand-average (±SEM) RMS difference value between the original and re-digitized data was 8.5±0.05 ADC counts per channel, or equivalently 20.8±0.12 µV.

**Table 1:** RMS difference values for all 10 patients' original versus re-digitized files when both the original and re-digitized files were collected on the same model of Cardiax ADC.

Channel								
Patient	I	II	CR1	CR2	CR3	CR4	CR5	CR6
**1H**	5.2	6.6	5.8	14.4	10.4	9.9	9.0	8.4
**2H**	2.8	6.8	4.2	13.6	9.1	9.6	8.3	8.0
**3H**	3.0	7.4	4.1	8.0	7.1	11.7	10.8	9.6
**4H**	7.1	8.0	7.4	13.2	9.9	9.2	9.4	9.2
**5H**	3.4	5.3	2.8	8.3	10.7	8.7	6.8	5.8
**1D**	2.2	3.5	3.3	6.0	8.0	5.1	4.7	4.7
**2D**	4.2	3.0	7.1	6.5	4.2	4.2	4.3	4.6
**3D**	3.2	3.2	5.6	8.3	7.0	5.7	5.5	5.5
**4D (LBBB)**	8.9	10.7	18.2	29.6	23.0	13.2	11.4	17.0
**5D (RBBB)**	12.5	13.8	9.7	13.9	15.1	15.2	16.7	14.7
**AVERAGES:**								
**H**	4.3	6.8	4.9	11.5	9.4	9.8	8.9	8.2
**D (no BBB)**	3.2	3.2	5.6	6.9	6.4	5.0	4.8	4.9
**D (with BBB)**	10.7	12.3	14.0	21.8	19.1	14.2	14.1	15.9

RMS: Root mean square, with RMS difference values expressed in analog to digital converter (ADC) counts, and with 1 ADC count = 2.44 µV.

Channel: the equivalent of leads I, II and the precordial electrodes as referenced to the right arm electrode (CR1-CR6).

H and D: Healthy and Diseased patients, respectively.

LBBB and RBBB: left and right bundle branch block (BBB), respectively.

Table 2 shows the estimated RMS difference values for each of the 8 independent ECG channels (PQRST) when the re-digitized data were instead collected on an ADC (i.e., CorScience's) that was different from the ADC (Cardiax's) used to collect and store the original data. Under these circumstances, the grand-average RMS difference values between the original and re-digitized data was 11.6±0.08 ADC counts per channel, or equivalently 28.4±0.21 µV.

**Table 2:** RMS difference values for all 10 patients' original versus re-digitized files when the original files were collected on a Cardiax ADC and the re-digitized files on a CorScience ADC.

Channel								
Patient	I	II	CR1	CR2	CR3	CR4	CR5	CR6
**1H**	5.3	6.9	6.2	10.4	9.5	9.5	9.2	8.6
**2H**	6.6	11.1	8.6	12.8	17.6	13.6	12.3	11.4
**3H**	8.3	9.7	8.7	15.7	11.3	13.6	12.0	11.2
**4H**	4.6	6.6	6.0	10.6	8.1	8.5	8.6	7.6
**5H**	6.3	9.4	7.1	9.9	12.7	11.1	9.8	8.7
**1D**	6.2	8.9	8.1	11.2	10.8	10.6	9.8	8.5
**2D**	11.5	11.6	13.2	13.9	15.2	15.7	15.4	13.2
**3D**	6.3	5.9	6.1	8.5	9.0	10.6	10.8	8.4
**4D (LBBB)**	10.8	18.3	21.5	37.3	27.8	17.3	19.4	26.3
**5D (RBBB)**	12.7	14.5	11.6	13.5	15.4	16.4	18.1	15.4
**AVERAGES:**								
**H**	6.2	8.7	7.3	11.9	11.8	11.3	10.4	9.5
**D (no BBB)**	8.0	8.8	9.1	11.2	11.7	12.3	12.0	10.0
**D (with BBB)**	11.8	16.4	16.6	25.4	21.6	16.9	18.8	20.9

RMS: Root mean square, with RMS difference values expressed in analog to digital converter (ADC) counts, and with 1 ADC count = 2.44 µV.

Channel: the equivalent of leads I, II and the precordial electrodes as referenced to the right arm electrode (CR1-CR6).

H and D: Healthy and Diseased patients, respectively.

LBBB and RBBB: left and right bundle branch block (BBB), respectively.

As can be surmised from Tables 1 and 2, there were no clear trends in the differences generated by the original versus re-digitized files in the healthy versus diseased subjects when the QRS interval was within a clinically normal range. However, as might be expected, the presence of either left (subject 4D) or right (subject 5D) bundle branch block, wherein the QRS interval is relatively prolonged and the total voltage relatively increased, tended to increase the differences between the voltages in the original versus re-digitized files.

### Qualitative validation: automated clinical diagnostic results

Table 3 shows the automated clinical diagnostic statements outputted by the commercial Cardiax software program for all 10 cases when both the original and re-digitized files were collected on the same model of Cardiax ADC. As can be surmised from Table 3, for all 10 cases under these circumstances, there were no differences in the clinical diagnostic statements outputted by Cardiax for the original versus the re-digitized files.

**Table 3:** Automated clinical diagnostic statements outputted by the Cardiax algorithm for the original versus re-digitized files when both files were collected on the same model of Cardiax ADC.

Patient	Original File	Re-digitized file
1H	No signs of abnormalities given the patient's age	No signs of abnormalities given the patient's age
2H	Sinus rhythm; 1 premature sinus complex	Sinus rhythm; 1 premature sinus complex
3H	Corresponds to the following pathological abnormality: undetermined rhythm	Corresponds to the following pathological abnormality: undetermined rhythm
4H	No signs of abnormalities given the patient's age	No signs of abnormalities given the patient's age
5H	No signs of abnormalities given the patient's age	No signs of abnormalities given the patient's age
1D	Sinus rhythm; suggests the following possible abnormality: left atrial enlargement	Sinus rhythm; suggests the following possible abnormality: left atrial enlargement
2D	Sinus rhythm; corresponds to the following pathological abnormality: with first-degree AV block (Long PQ); undetermined variation: T wave abnormality	Sinus rhythm; corresponds to the following pathological abnormality: with first-degree AV block (Long PQ); undetermined variation: T wave abnormality
3D	No signs of abnormalities given the patient's age	No signs of abnormalities given the patient's age
4D	Sinus rhythm; corresponds to the following pathological abnormality: left bundle branch block	Sinus rhythm; corresponds to the following pathological abnormality: left bundle branch block
5D	Sinus rhythm; corresponds to the following pathological abnormalities: 1 premature ventricular complex; right bundle branch block	Sinus rhythm; corresponds to the following pathological abnormalities: 1 premature ventricular complex; right bundle branch block

H and D: Healthy and Diseased patients, respectively.

**Figure 3:**
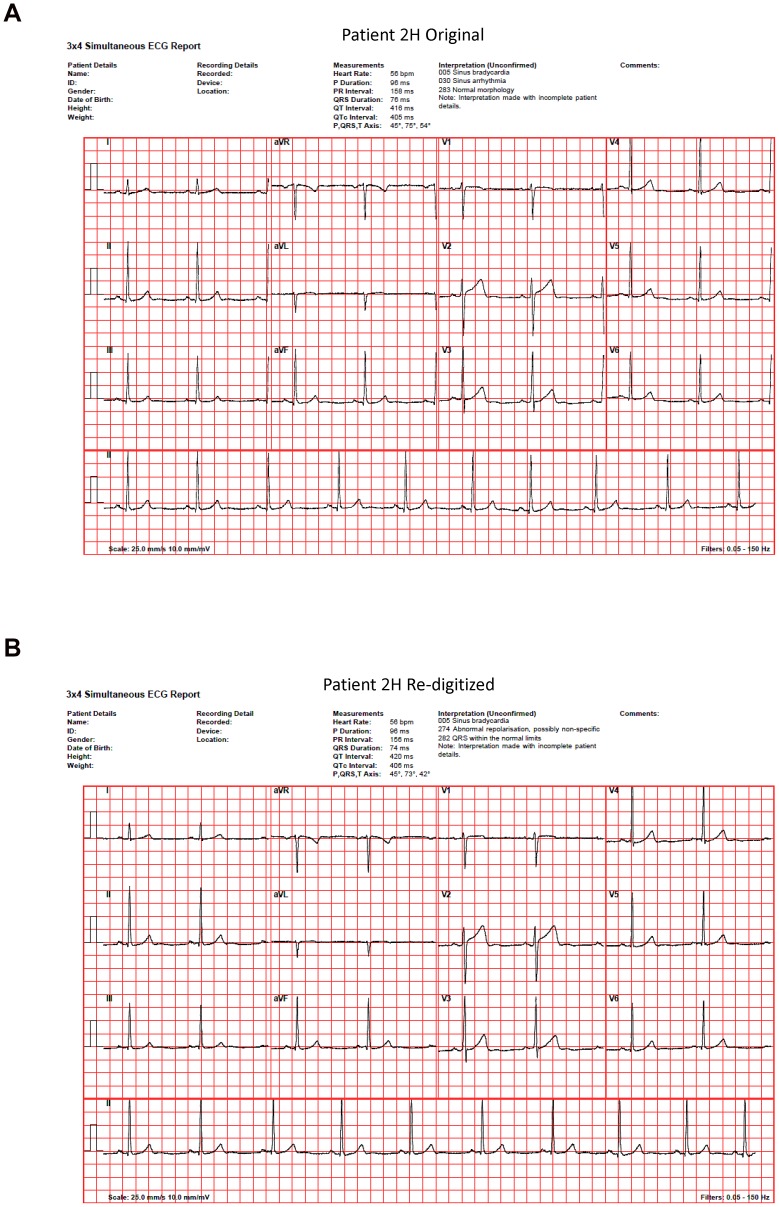
“Worst case” result from a qualitative standpoint. Original (**A**) and re-digitized (**B**) 12-lead ECG tracings from patient 2H as interpreted by the Leuven automated diagnostic algorithm when a Cardiax ADC was used to collect the original data and a CorScience ADC the re-digitized data. This was the only file amongst the 10 tested wherein a minor change was elicited in the automated interpretation of the re-digitized compared to the original file. This minor change occurred only when using the Leuven algorithm (a corresponding change did not occur for the automated interpretation when using the Cardiax algorithm under any circumstances), and occurred regardless of whether the re-digitized data were collected on a CorScience or Cardiax ADC. Note also the modest change in DC offset (which may have been a key contributor to the slight change in the automated interpretation) as well as the very minor differences between (A) and (B) in some intervals, axes and voltages as automatically determined.

**Figure 4:**
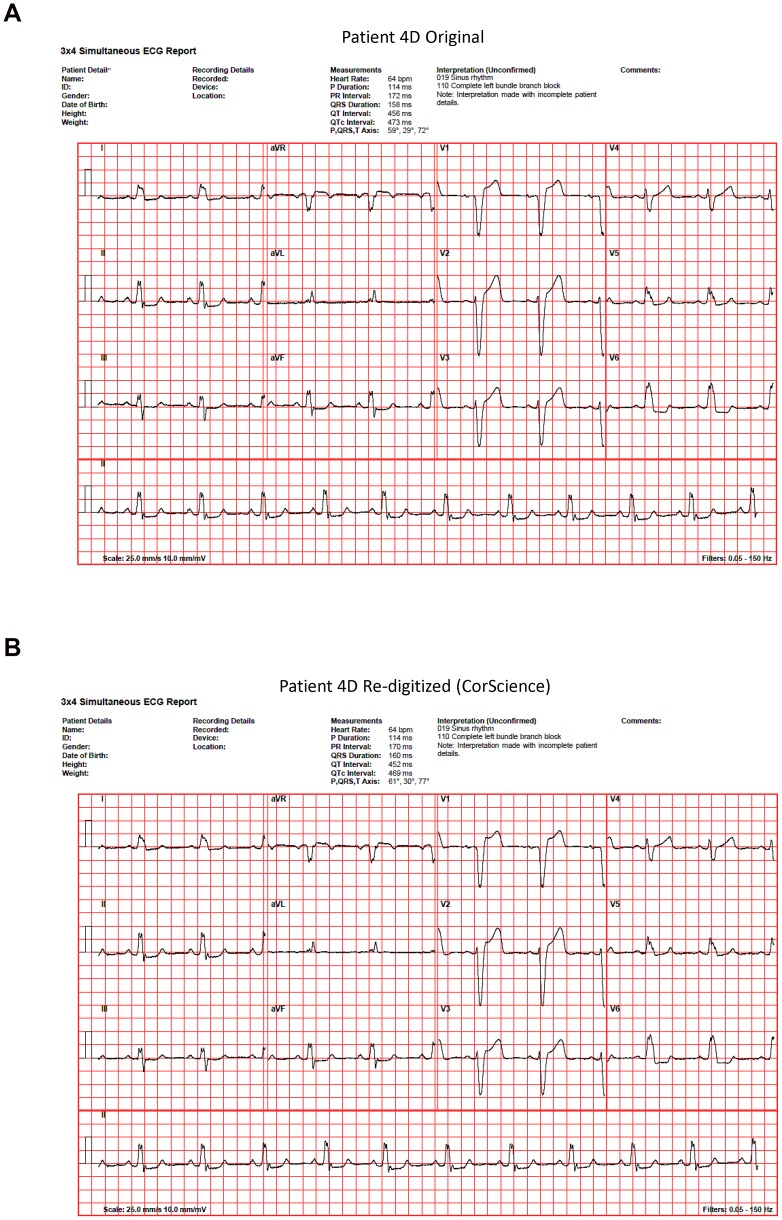
“Worst case” result from a quantitative standpoint. Original (**A**) and re-digitized (**B**) 12-lead ECG tracings from patient 4D as interpreted by the Leuven automated diagnostic algorithm when a Cardiax ADC had been used to collect the original data and a CorScience ADC the re-digitized data. This patient has a left bundle branch block and the results shown in (B) represent the quantitative “worst case” encountered during the study inasmuch as the voltage differences between original and CorScience re-digitized files were the largest noted overall (Table 2). Consistent with the data in Table 2 (and in Table 1), the most pronounced differences between this patient's original and re-digitized files occurred in his leads V1–V3 (i.e., emanating from channels CR1–CR3), where, in the CorScience re-digitized compared to the original file, a slight additional concavity could also be *visually noted* in the ST segments.

Table 4 shows the automated clinical diagnostic statements outputted by the commercial Leuven software program for all 10 cases when the original files were collected on the Cardiax ADC and when the re-digitized files were collected on either the Cardiax or CorScience ADC (i.e., the ultimate interpretive results from the Leuven program were the same under both of the above circumstances). Under either of these circumstances, the automated diagnostic statements outputted by the Leuven program for the original versus the re-digitized files differed for only one case (i.e., for healthy patient 2H). Specifically, within the Leuven program, criteria for “abnormal repolarization, possibly non-specific” were triggered for patient 2H's re-digitized file whereas such criteria were not triggered for this same patient's original file. While it is unclear whether this minor difference in the Leuven algorithm's automated interpretation would have made any clinical difference (we suspect not), the original and re-digitized ECGs for this patient as interpreted by the Leuven algorithm are shown in Figure 3. Both Figure 3 and Figure 4 (which shows our corresponding “worst-case comparison” between original and re-digitized files as quantified by the greatest differences in RMS values; patient 4D) also aptly demonstrate the minor differences that typically occurred between all original versus re-digitized files with respect to the various electrocardiographic axes, intervals, and voltages that were outputted by the automated interpretive software.

**Table 4:** Automated clinical diagnostic statement(s) outputted by the Leuven algorithm for the original vs. re-digitized files when the original file was collected on a Cardiax ADC and the re-digitized file on either a Cardiax or CorScience ADC.

Patient	Original File	Re-digitized file
1H	Sinus arrhythmia; normal morphology	Sinus arrhythmia; normal morphology
2H	Sinus bradycardia; *normal morphology*	Sinus bradycardia; *abnormal repolarization, possibly non-specific; QRS within the normal limits*
3H	Sinus arrhythmia; abnormal repolarization, possibly non-specific; QRS within the normal limits	Sinus arrhythmia; abnormal repolarization, possibly non-specific; QRS within the normal limits
4H	Sinus bradycardia; normal morphology	Sinus bradycardia; normal morphology
5H	Normal sinus rhythm; normal morphology	Normal sinus rhythm; normal morphology
1D	Normal sinus rhythm; normal morphology	Normal sinus rhythm; normal morphology
2D	Sinus rhythm with first-degree AV block; left atrial hypertrophy; abnormal repolarization, possibly non-specific; QRS within the normal limits	Sinus rhythm with first-degree AV block; left atrial hypertrophy; abnormal repolarization, possibly non-specific; QRS within the normal limits
3D	Normal sinus rhythm; possible inferior infarction, probably old	Normal sinus rhythm; possible inferior infarction, probably old
4D	Sinus rhythm; complete left bundle branch block	Sinus rhythm, complete left bundle branch block
5D	Sinus rhythm; ventricular extrasystole(s); ventricular extrasystole(s) with full compensation; complete right bundle branch block	Sinus rhythm; ventricular extrasystole(s); ventricular extrasystole(s) with full compensation; complete right bundle branch block

H and D: Healthy and Diseased patients, respectively. The single difference noted in the automated diagnostic interpretations (original versus re-digitized) is shown in italics (file 2H).

## Discussion

**Figure 5:**
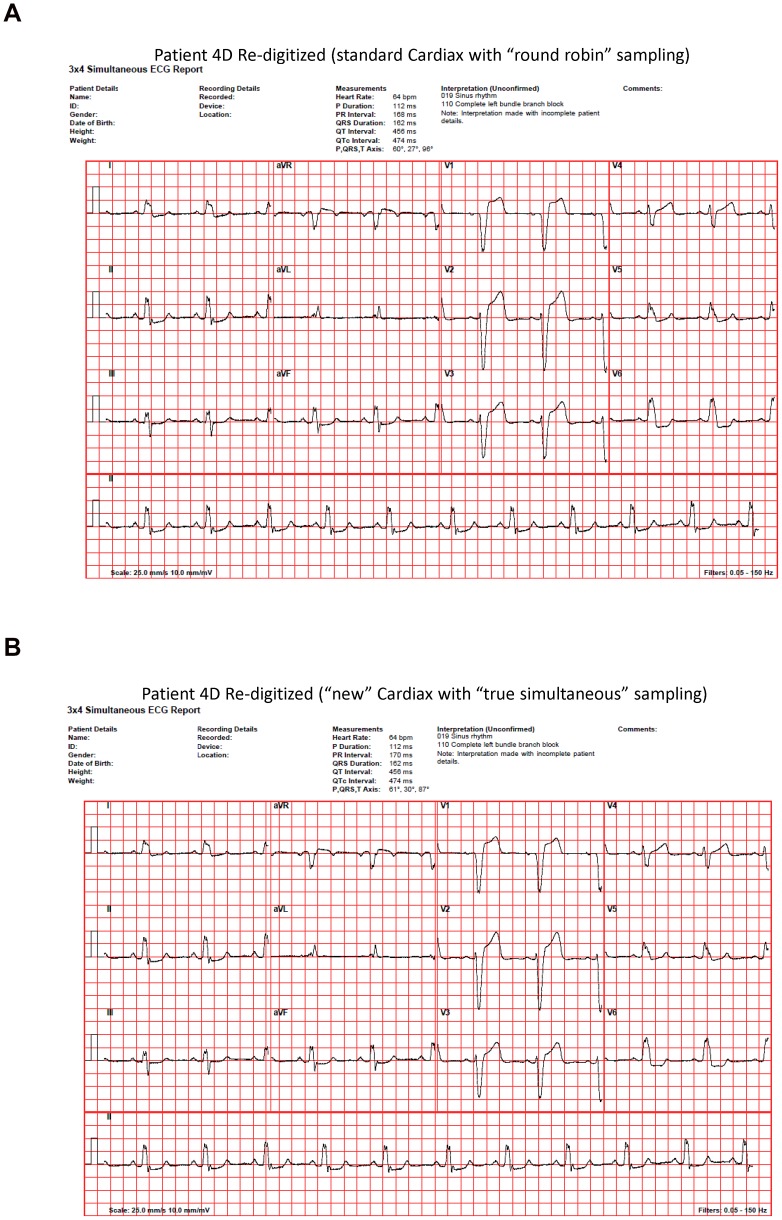
Effect of “true simultaneous” sampling. (**A**) The study's standard “round-robin sampled” Cardiax-re-digitized file for the same patient 4D with a left bundle branch block whose original file is shown in Figure 4A. Possibly due in part to the higher sampling rate at Cardiax's compared to CorScience's ADC (i.e., 1000 Hz rather than 500 Hz), the visual differences in this patient's leads V1–V3 between the Cardiax re-digitized and original file are perhaps slightly less apparent than those between the CorScience re-digitized and original file as observed in Figure 4. (**B**) When using for re-digitization a just-released new Cardiax device briefly loaned to us after our formal study's completion that employs “true simultaneous” sampling via incorporation of Texas Instruments' ADS1298 chip, the visual differences in this same patient's V1–V3 complexes essentially “disappear” in conjunction with a ∼2–3 fold reduction in the RMS difference values for channels CR1, CR2 and CR3 to 9.4, 9.4 and 11.7 ADC counts, respectively. Compare these results to the corresponding results for CR1–CR3 for this patient as shown in Tables 1 and 2 when “non true-simultaneous sampling” was used for re-digitization.

Our results suggest that the system described herein can currently reproduce original analog signals from stored 12-lead ECG data files with a degree of fidelity likely sufficient for most clinical needs. In our formal study, one possible exception might have been when the system was used to reconstruct files that had bundle branch blocks, i.e., wherein quantitative reconstruction errors were at their highest (Tables 1 and 2 and Figure 4). In relation to this, it should be noted that the Cardiax and CorScience ADCs employed in our study use, like the majority of ADCs incorporated into other commercially available ECG devices, known non-optimal methods of sampling that implement “time interleaving”. Importantly, such methods alone, whether they implement “round robin” (e.g., Cardiax) or “pseudo-simultaneous” (e.g., CorScience) sampling, may introduce certain subtle distortions into any digitized data (and thus also into any re-digitized data) [9], [10]. For the first time, some of the newest ECG devices just introduced into the market now incorporate ADCs employing a more truly simultaneous method of sampling, made possible by new chips like Texas Instruments' ADS1298. Thus digital data collected on devices employing such new chips may, with even greater fidelity, be re-convertible back to the original analog. Even more importantly, our own preliminary testing with one of these new devices (a new Cardiax device that now incorporates the ADS1298) for ultimate reconstruction rather than original data collection suggests that machines like it will notably further improve the quality of *re-digitization* (Figure 5). The substantial reduction in the quantitative RMS error values noted in Figure 5B (2–3 fold compared to the values shown in Tables 1 and 2) when using a device with “true simultaneous sampling” for ultimate re-digitization/reconstruction therefore provides evidence that files with bundle branch blocks can also be reproduced with clinically acceptable accuracy as long as the specifications of the ADC in the final recipient machine are sufficiently technologically advanced.

While our results further corroborate the utility of the one commercial device that to our knowledge currently applies DAC to ECG in a clinical setting–i.e., the aforementioned LifeSync® device utilized in hospitals—the overall greater utility, flexibility, universality, more open format, and “readiness for cloud computing” of our system potentially open up several new avenues for more widespread use of DAC devices in clinical electrocardiography. Specifically, without requiring manufacturer-adjudicated digital access into any automated interpretive functionality, systems such as ours might eventually allow for all of the following: *1*) rapid second opinions from any number of automated interpretive programs, e.g., for difficult-to-interpret 12-lead ECGs and rhythms (not only locally, but also from dedicated remote central or cloud-based servers; *2*) use of less expensive (i.e., commodity-grade) 12-lead ECG front ends (ADC hardware) in impoverished or underserved areas, because subsequent DAC will always permit use of any preferred (or any otherwise prohibitively-expensive) ECG machine or interpretive program only singly, on the back end; *3*) use of less bulky ECG front ends during space flight or in other terrestrially remote environments; *4*) improved performance of all automated ECG analytical software programs through the implementation by manufacturers of those “interpretive lessons learned” that will be more rapidly ascertainable to them both through internal testing and through objective competitions enabled by the DAC; *5*) better within-hospital consistency of automated ECG interpretations, e.g., when ECG machines from multiple different manufacturers are used in any single institution; and *6*) better across-study consistency when large digital ECG databases are analyzed in epidemiological studies, as the DAC should allow for the same analytical programs to be used, when desired, across all such large studies, even when different collaborating groups don't all possess the same hardware and software.

It should be reemphasized that the only prerequisite for the use of the described system is that the format of the original digital data must be known – i.e., to permit pre-conversion into an optimal, open digital format for DAC such as the one described in Appendix S1—a functionality easily performed by either integrated or secondary software tailored to make such conversions. Once the data are converted to the optimized format either locally or remotely, then the hardware aspect of the system can also be readily employed either locally or remotely to convert the digital data to analog and then in turn to stream the analog data into any desired 12-lead ECG machine.

### Limitations

The main limitation to this first proof-of-concept study is that it constitutes a limited initial validation wherein we have only formally analyzed a small number of stored digital files using hardware from two different ECG manufacturers. While non-formally we have also successfully employed the DAC to input data originally collected on several larger ECG manufacturers' machines into receiving machines from other large ECG manufacturers, future studies will ideally include the formal analyses of a larger number of files and electrocardiographic conditions and machines, and/or focus especially on those subtle ECG conditions that might be most susceptible to being “masked” (or to being spuriously introduced) in re-digitized recordings.

## Conclusion

In conclusion, we have built a new system for digital to analog conversions of 12-lead ECG data and partially validated it through study of original versus re-digitized 12-lead ECG recordings from five healthy and five diseased individuals. Our results suggest that in the near future, systems like this will allow for rapid automated second opinions on difficult-to-interpret 12-lead ECGs and rhythms, for improvements to all manufacturers' automated 12-lead ECG interpretations, and for use of less expensive (or less bulky) ECG hardware front ends in impoverished, remote and other areas. Moreover the mere existence and availability of this new DAC system may provide an important stimulus to increase the willingness of all ECG manufacturers to participate in potentially more convenient, purely digital, multi-manufacturer interchanges for “automated 12-lead ECG second opinions”, to the further benefit of both patients and clinicians.

## Supporting Information

Appendix S1 **The optimized data format used with the system.**
(DOC)

Appendix S2 **Hardware and software configurations of the prototype, including calibration and data processing.**
(DOC)
