# Supplementary material for: New System for Digital to Analog Transformation and Reconstruction of 12-Lead ECGs
Source: PLoS One. 2013 Apr 11;8(4):e61076. doi: 10.1371/journal.pone.0061076 (PMC3623879; doi:10.1371/journal.pone.0061076)
Supplement: Appendix S1 — The optimized data format used with the system. (DOC) [file pone.0061076.s001.doc]

*Header:* Stored or other ECG data to be converted back to analog need not have a header.

*Sample value:* Although this can theoretically vary without great consequence, the sample value in the format we use is a 16-bit signed integer, ranging from +2047 to -2048, in Intel byte order, meaning low byte first (little endian).

*Format:* The DAC device presently assumes that the incoming digital data will be in binary format, as one would obtain directly from a multiplexed ADC. The specific format we use is presented on a sample-by-sample basis further below. However, as noted in the text, it should first be understood that the preferred digital format is one wherein all given precordial electrodes are referenced not to Wilson’s central terminal but rather to a limb electrode, most conveniently to the right arm electrode, making our own preferred precordial channels the CR channels rather than the V leads. Right arm electrode-referenced precordial channels are ideal for a pre-DAC digital format because as described in the text, the repeat ADC (i.e., by any given 12-lead ECG device) that follows an appropriately configured DAC step will then naturally convert the so-formatted precordial channel data back to the V precordial lead format by using whatever scheme the given ECG manufacturer uses to accomplish that specific task on the instrumented human. The *Programming notes* below provide further background information on how a right arm electrode-referenced 12-lead ECG data format can be accomplished in software and applied either to ADC, or, as in our case, to an optimal digital format for pre-DAC.

*Programming notes:*

// 9+1 channels, e.g., from the electrodes on a patient:

// EL, EF, ER, EC1, EC2, EC3, EC4, EC5, EC6 (+N)

// EL: left arm electrode

// EF: left leg electrode

// ER: right arm electrode

// N: right leg electrode (reference neutral)

// ECi: chest (precordial) electrodes

// Measured (raw data): (8 channels): CL, CF, CR1, ..., CR6

// CL=EL-ER, CF=EF-ER, CR1=EC1-ER, ..., CR6=EC6-ER

// combined:

// I = EL-ER = CL

// II = EF-ER = CF

// III = EF-EL = (EF-ER)-(EL-ER) = CF-CL

// aVR = ER-(EL+EF)/2 = (2*ER-EL-EF)/2 = -((EL-ER)+(EF-ER))/2 = -(CL+CF)/2

// aVL = EL-(EF+ER)/2 = (2*EL-EF-ER)/2 = (2*(EL-ER)-(EF-ER))/2 = CL-CF/2

// aVF = EF-(EL+ER)/2 = (2*EF-EL-ER)/2 = (2*(EF-ER)-(EL-ER))/2 = CF-CL/2

// Vi = ECi-(EL+EF+ER)/3 = (3*ECi-EL-EF-ER)/3 = (3*(ECi-ER)-(EL-ER)-(EF-ER))/3 =

// = (ECi-ER)-((EL-ER)+(EF-ER))/3 = CRi - (CL+CF)/3 // i = 1...6

// The binary data are thus constituted by I, II, and CR1-6 as eight independent data channels

// Furthermore, Vi = CRi-(I+II)/3 and thus CRi = Vi+(I+II)/3

// Note that CRi = ECi if ER = 0, an important part of the basis for the optimized format

Given the above information and definitions, the optimized data format ultimately used with (input into) the DAC device can be represented as follows:

**Sample (0) :** Channel 1 (= CL = lead I) (2-bytes) Byte 0-1

Channel 2 (= CF = lead II) (2-bytes) Byte 2-3

Channel CR1 (2-bytes) Byte 4-5

Channel CR2 (2-bytes) Byte 6-7

Channel CR3 (2-bytes) Byte 8-9

Channel CR4 (2-bytes) Byte 10-11

Channel CR5 (2-bytes) Byte 12-13

Channel CR6 (2-bytes) Byte 14-15

**Sample (1) :** Channel 1 (= CL = lead I) (2-bytes) Byte 16-17

Channel 2 (= CF = lead II) (2-bytes) Byte 18-19

Channel CR1 (2-bytes) Byte 20-21

Channel CR2 (2-bytes) Byte 22-23

Channel CR3 (2-bytes) Byte 24-25

Channel CR4 (2-bytes) Byte 26-27

Channel CR5 (2-bytes) Byte 28-29

Channel CR6 (2-bytes) Byte 30-31

**Sample (n) :**  etc.
