# Supplementary material for: New System for Digital to Analog Transformation and Reconstruction of 12-Lead ECGs
Source: PLoS One. 2013 Apr 11;8(4):e61076. doi: 10.1371/journal.pone.0061076 (PMC3623879; doi:10.1371/journal.pone.0061076)
Supplement: Appendix S2 — Hardware and software configurations of the prototype, including calibration and data processing. (DOC) [file pone.0061076.s002.doc]

*Hardware configuration.* Our initial hardware prototype consisted of a 4-slot CompactDAQ chassis (cDAQ-9174, National Instruments, Austin, TX) fitted with a multi-channel, 16-bit, ±10 V analog output module (NI 9264); custom cables made with D-sub connectors; and a voltage divider box. For reasons related to both cost and portability, we chose to build our initial prototype around a cDAQ rather than around a larger, more expensive DAC system that must be placed inside a desktop computer.

In the prototype, digital data in the optimized format are input via the USB interface to dedicated channels of the DAC system which in turn outputs the corresponding analog signals to commensurately dedicated pins. The associated voltage travels through a cable via a DC-37 socket connector to a voltage divider box via a DB-25 connection. The voltage divider box is constructed with high precision 100 Ω and 100 kΩ metal film resistors to decrease the voltage of each lead by a factor of 1001:1. The final ECG analog signals are then output by a DB-15 connector that can be immediately utilized by any ECG machine already employing the DB-15 IEC standard, or subsequently utilized by any ECG machine of the user’s choice through use of a simple DB-15 to button receptacle (or to banana receptacle) adapter box.

*Calibration of the voltage divider.* Although ultra-high precision resistors with ±0.1% resistance tolerance are used in our prototype, a slight variation in resistance values can introduce error into data conversion. Therefore, each voltage divider’s calibration factor was first determined and implemented in software. To find the calibration factors, a constant 3V signal was passed through each pin of the voltage divider box and sent to a high-sensitivity, 7½ Digit Nano Volt/Micro Ohm Meter (Agilent Technologies, Model 34420A). For each channel, the original value of 3V was divided by the voltage output from the voltage divider box to calculate the calibration factors. A text file containing the calibration factors specific to the given individual DAC device was then constructed and is implemented inside the system-associated software program.

*Software configuration.* The software portion of the prototype system is a PC-based application programmed in C language (LabWindows/CVI version 8.0, National Instruments, Austin, TX). It includes a basic user interface from which the user can first choose the system-specific calibration text file for the given DAC device, and then also a program mode such as “one run-through” versus “continuous cycling”. If for a stored ECG data file the “one run-through” mode is selected before data conversion is started, then the actual conversion will stop when the end of the file is naturally reached and all data in the file have been converted to analog. If instead a “continuous cycling” mode is selected, then the program will continuously loop through (replay) the file and continue to convert to analog until a “stop” button is activated or the program is closed. At this point, a new digital file or data stream to be processed can then be chosen.

*ECG data processing.* The program presently accepts digital data in the optimized binary format outlined in Appendix S1. ECG data in different known formats not optimal for DAC can be pre-converted into the optimized format prior to system use. Once the user selects within the program the optimally formatted digital data that he/she desires to transform to analog, the program opens the specified data or file, reads 400 samples per each of the 8 channels into a buffer and then interpolates those samples to double the size of the buffer. The calibration text file is also read into an array. The data in the buffer are then separated into a 2D array with 8 channels, and multiplied by the calibration factor specific to each channel with the input ADC data scaled so that 5 mV at the original ADC is 5 V at the DAC output. The original data are also displayed on an onscreen waveform strip chart as they are sent to the DAC to be converted to analog voltage. This entire general process of DAC (and then repeat ADC) is summarized in Figure 2 of the text.
